# Using Goal- and Grip-Related Information for Understanding the Correctness of Other’s Actions: An ERP Study

**DOI:** 10.1371/journal.pone.0036450

**Published:** 2012-05-11

**Authors:** Michiel van Elk, Roel Bousardt, Harold Bekkering, Hein T. van Schie

**Affiliations:** 1 Donders Institute for Brain, Cognition and Behaviour, Radboud University Nijmegen, Nijmegen, The Netherlands; 2 Laboratory of Cognitive Neuroscience, Brain Mind Institute, École Polytechnique Fédérale de Lausanne, Lausanne, Switzerland; 3 Behavioural Science Institute, Radboud University Nijmegen, Nijmegen, The Netherlands; University College London, United Kingdom

## Abstract

Detecting errors in other’s actions is of pivotal importance for joint action, competitive behavior and observational learning. Although many studies have focused on the neural mechanisms involved in detecting low-level errors, relatively little is known about error-detection in everyday situations. The present study aimed to identify the functional and neural mechanisms whereby we understand the correctness of other’s actions involving well-known objects (e.g. pouring coffee in a cup). Participants observed action sequences in which the correctness of the object grasped and the grip applied to a pair of objects were independently manipulated. Observation of object violations (e.g. grasping the empty cup instead of the coffee pot) resulted in a stronger P3-effect than observation of grip errors (e.g. grasping the coffee pot at the upper part instead of the handle), likely reflecting a reorienting response, directing attention to the relevant location. Following the P3-effect, a parietal slow wave positivity was observed that persisted for grip-errors, likely reflecting the detection of an incorrect hand-object interaction. These findings provide new insight in the functional significance of the neurophysiological markers associated with the observation of incorrect actions and suggest that the P3-effect and the subsequent parietal slow wave positivity may reflect the detection of errors at different levels in the action hierarchy. Thereby this study elucidates the cognitive processes that support the detection of action violations in the selection of objects and grips.

## Introduction

An important question is how we understand the correctness of other’s actions. For instance, when the person in front of you buys a ticket at a vending machine in a railway station, it can be quite annoying if you see him making an error (e.g. trying to put his credit card in the wrong slot) and you may want to point out the correct action. On the other hand, in case you observe someone performing a novel action (e.g. checking in luggage at a novel baggage-drop-off system), you can learn from the other’s errors. As these examples illustrate, error detection in action observation enables both cooperative behavior and observational learning. In addition, error detection is pivotal for competitive behavior as well. For instance in many games and sports we take advantage of detecting action slips of our opponent.

Several neural mechanisms have been proposed to underlie the detection of action errors. Some studies have shown the involvement of mirror neuron areas, such as the inferior parietal lobe (IPL) and the premotor cortex (PM) in the observation of erroneous actions [1], [2]. Related to this, other studies have shown that the observation of an incorrect action results in a stronger desynchronization and subsequent rebound in the beta frequency band, supposedly reflecting a stronger activation of sensorimotor areas [3], [4], [5]. Both the execution and the observation of errors have been associated with the error-related negativity (ERN), an early negative deflection in the EEG likely originating from the anterior cingulate cortex (ACC) [6], [7], [8]. Most studies on error detection have used relatively low-level errors and stimuli (e.g. observing a hand pressing the left instead of the right button), leaving open the question how we understand the (in)correctness of other’s actions in everyday situations.

Event-related potential (ERP) studies using real-world stimuli have shown that the observation of action errors (e.g. watering the table instead of the plant) resulted in an enhanced P3-component and a subsequent parietal positivity [9], [10]. However, the functional significance of these findings is not entirely clear. Some authors have suggested that the stronger P3 for incorrect actions reflects a *monitoring mechanism* supporting the detection of action slips [9]. In contrast, other studies, using relatively simple detection tasks, suggest that the P3 reflects a *reorienting response*, following the detection of an unexpected stimulus [11], [12]. Interestingly, a similar parietal positive slow wave effect has been found in association with the execution of goal-directed actions, when subjects were required to actually reach towards real-world objects [13], [14]. More specifically, van Schie and Bekkering (2007) reported a parietal positive slow wave for planning and executing movements that was found maximal at the moment of object grasping. Accordingly, one alternative interpretation of the parietal positivity for the observation of incorrect actions is that it reflects a *representation of the hand-object interaction*.

The different interpretations of the P3 effect and the subsequent slow wave positivity in association with the observation of action errors may be related to the fact that previous studies did not clearly distinguish between different levels of action errors. That is, action correctness can be defined at different levels in the action hierarchy. For instance, an action can be directed towards an incorrect object (e.g. grasping a cup instead of a coffee pot) or an object can be grasped in an incorrect way (e.g. grasping a coffee pot with a grip that does not afford pouring coffee). Several behavioral studies have suggested a dominance of processing goal- over grip-related information in understanding the correctness of others’ actions [15], [16], [17]. For instance, participants were faster in judging the correctness of an action, when asked to focus on the goal of the action than when instructed to attend to the grip of the action [16]. This finding is in line with the hierarchical view of the motor system, according to which our ability to perform complex actions relies on the hierarchical organization of the motor system [18], [19], [20].

The hierarchical view of the action system implies that the processing of goal-errors is faster than the processing of grip errors. Accordingly, the P3 and subsequent slow wave positivity may reflect different aspects of observed actions. In the present study we investigated the hypothesis that the P3-effect associated with the observation of action errors reflects a reorienting response following the identification of actions directed towards the incorrect object. In contrast, the later parietal positive slow wave associated with the observation of action errors [9], [10] may reflect the detection of a grip-error (i.e. incorrect handgrip applied for grasping an object). To test this hypothesis we used an experimental paradigm in which participants were required to judge the correctness of actions involving everyday objects. Participants observed action sequences that involved two objects: a tool (e.g. a bubble blower) and a target object (e.g. a soap bottle). The object pairs implied a specific action sequence and a specific way of grasping that could be inferred based on the conceptual properties of the objects (e.g. typically a bubble blower is grasped at the handle to soak it subsequently in the soap bottle).

**Figure 1:**
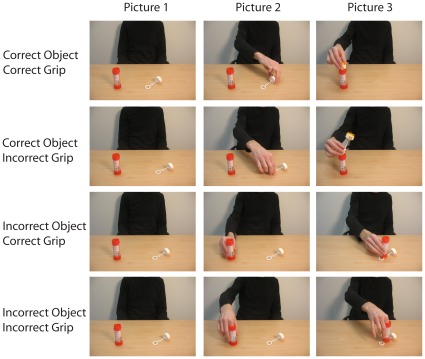
Example stimuli used in the experiment. For each object pair an action sequence was constructed, consisting of 3 action snapshots (left column, middle column and right column). The correctness of the object grasped and the grip applied to the object were independently manipulated, resulting in action sequences representing an actor (1) grasping the correct object with a correct grip (upper row), (2) grasping the correct object with an incorrect grip (2^nd^ row), (3) grasping the incorrect object with a correct grip (3^rd^ row) or (4) grasping the incorrect object with an incorrect grip (bottom row).

In the experiment the correctness of the object grasped and the grip applied to the object were independently manipulated (see Figure 1). This manipulation allowed us to investigate whether the P3- and the parietal slow wave effect were selectively modulated by object and/or grip-violations. *Object correctness* was defined with respect to whether the tool (e.g. the bubble blower) or the target object (e.g. the soap bottle) was grasped first. In order to combine both objects in a meaningful action sequence, the tool needs to be grasped first and therefore by definition tools have a higher probability of being grasped first than target objects. Thus, an object error was defined as grasping the target object first instead of the tool. *Grip correctness* was defined with respect to the appropriateness of the grip applied to the object for actually interacting with the object. For instance, grasping a tool with an incorrect grip does not allow using the tool in combination with the target object (e.g. grasping the bubble blower at the opening does not afford bubble blowing).

Based on previous studies we expected that the observation of incorrect actions would be associated with a stronger P3 component and a subsequent parietal positive slow wave [9], [10]. In addition, following the notion that actions are processed in a hierarchical fashion we expected that object errors would be detected earlier than grip errors and should result in a reorienting response, as reflected in a stronger P3-effect [11], [12]. The processing of grip errors may be associated with a reorienting response as well, followed by a relatively late parietal slow wave positivity, which could reflect the detection of an incorrect hand-object interaction.

## Materials and Methods

### Subjects

For pretesting the stimuli 19 participants were tested (4 males, mean age = 22.4 years, SD = 4.9 years). In the EEG experiment, 19 participants were tested (6 males, mean age = 21.2 years, SD = 3.7 years), who had not participated in the pre-test. Data from five participants were discarded from analysis, due to insufficient artifact-free trials (i.e. less than 30 trials remaining per condition). All participants were students at the Radboud University Nijmegen who participated for course credits or an experimental remuneration. All participants were right-handed and had normal or corrected-to-normal vision. All participants gave written informed consent prior to the experiment. The study was approved by the local ethics committee (Commissie Mens Gebonden Onderzoek Regio Arnhem-Nijmegen) and was conducted in accordance with the declaration of Helsinki.

### Stimuli

As stimuli we used pictures representing an actor sitting behind a table, on which two different objects were placed. In total 48 different object pairs were used that each implied a specific action sequence (see Table 1). Each object pair consisted of a tool and a target object (e.g. a bubble blower and a soap bottle, a sugar bowl and a cup; for example stimuli, see Figure 1). For each object pair, an action sequence was constructed, consisting of 3 action snapshots representing: (a) an actor sitting behind the table on which the objects were placed, (b) the actor grasping one of the objects and (c) the actor moving one object to the other object (see Figure 1 for example stimuli). The correctness of the object grasped and the grip applied to the object were independently manipulated. Accordingly, for each object pair 4 different action sequences were taken, representing an actor (1) grasping the correct object with a correct grip, (2) grasping the correct object with an incorrect grip, (3) grasping the incorrect object with a correct grip and (4) grasping the incorrect object with an incorrect grip (see Figure 1). Grip errors were defined as grasping the object at the incorrect part that would not afford its use in an action sequence (e.g. it is not possible to use a bubble blower when it is grasped at the opening instead of the handle and putting one’s finger in the soap bottle in order to grasp it also does not afford using the soap). In addition, for each object pair the location of the objects was switched to avoid the correct object from being always on the ipsilateral side of the movement. Thus, for each object pair 8 different action sequences were constructed according to an Object (Correct vs. Incorrect) x Grasp (Correct vs. Incorrect) x Location (Ipsilateral vs. Contralateral) design. It should be noted that the actions associated with each object pair were matched for difficulty. That is, each tool could be used in combination with the target object by means of a simple grasping- and transport-movement. For instance, the lid was already removed from the teapot, so as to allow the simple insertion of the teabag in the teapot. Although the analysis focused only on the onset of the second picture, the inclusion of a third picture representing the outcome of the action was deemed necessary, to provide a context for making judgments about the correctness of the object grasped and the grip applied to the object.

**Table 1:** Object pairs used in the EEG experiment.

	Tool	TargetObject		Tool	TargetObject
1	battery	digital camera	25	knife	butter
2	bottle opener	beer bottle	26	ladle	soup bowl*
3	brush	paint	27	lightbulb	lamp
4	bubble blower	soap	28	lighter	candle*
5	buttered knife	bread	29	magnifying glass	stamps
6	cd	cd player	30	paint tube	paper
7	cd	cd case	31	paper	perforator*
8	chalk	blackboard	32	paper	letter tray
9	coffee	coffee filter	33	pencil	paper
10	coffee filter	filter holder	34	pen	notebook
11	cola can	empty glass	35	pizza knife	pizza*
12	cover	pan	36	power cord	socket
13	creditcard	wallet	37	sprinkles	bread
14	dish brush	wash tub	38	stamp	stamp pad
15	drumstick	drum	39	stick	xylophone
16	egg	egg holder	40	straw	glass
17	eraser	blackboard	41	sugar bowl	cup
18	eraser	line drawing	42	sunglasses	glasses case
19	flour	kitchen balance	43	teabag	teapot
20	garbage	trash bin	44	thermos	mug*
21	glue	paper	45	toothpaste	toothbrush*
22	iron	shirt	46	water can	glass
23	kettle	stand	47	weight	kitchen balance
24	key	lock	48	whisk	bowl

The left column represents tools that had a high probability of being grasped first. The right column represents target objects towards which the tools could be moved. Object pairs marked with an asterisk were used as practice trials and were not included in the main EEG experiment.

To test for possible ambiguities in the action sequence implied by the object pairs a pre-test was conducted. Participants were presented with a picture representing each object pair without an action and were required to predict which object would probably be grasped first, by means of a left/right button press. After each picture participants rated the predictability of the object pair on a 7-point Likert scale. In an item analysis the percentage of object pairs that differed from the pre-specified assignment of objects to tools and targets was 10.9% (SD = 14.4) and the average reaction time was 1195 ms (SD = 221 ms). Overall, participants were well able to correctly predict which object would be grasped first (mean predictability = 2.3, SD = .63; 1 = very predictable, 7 = very unpredictable). A correlation was observed between reaction times and the predictability rating, r = .504, p<.001, reflecting that objects pairs that were responded to slowest also were classified as being less predictable. To obtain a reliable measure of the predictability of the object pairs, a factor analysis was conducted using principal component analysis [21]. One factor accounted for 66% of the variance observed in the error rates, the reaction times and the predictability rating. On the basis of the factor loadings six object pairs that were highly unpredictable (factor loading >1) were excluded from the stimulus set, leaving 42 object pairs in the final stimulus set used for the EEG experiment. The 6 object pairs that were not included were used as practice trials for the EEG experiment.

### Experimental Design and Procedure

**Figure 2:**
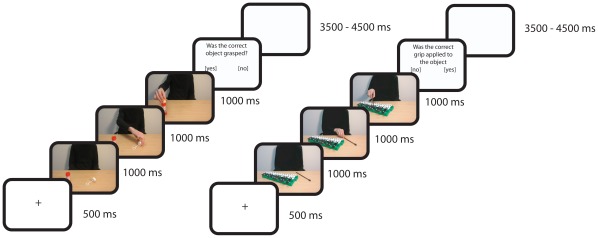
Schematic overview of a trial sequence. Each trial started with a fixation cross, after which the action sequence was presented, consisting of 3 action snapshots. Following the action sequence either an object question (left side) or a grip question (right side) could be presented. Mapping of the response buttons (left/right) varied between trials and was presented below the question. After the subject responded a blank screen was presented.

Each trial started with the presentation of a fixation cross for 500 ms. Next the action sequence was presented for 3000 ms (1000 ms per action snapshot). Each action sequence was followed by either an object question (“was the correct object grasped?”) or a grip question (“was the correct grip applied to the object?”). Object questions and grip questions were randomly presented, in order to ensure processing of both object- and grip-related information during each trial. Participants were required to respond to the question by pressing the left or the right button of a button box with their right hand. The mapping of response buttons (yes/no) was varied between trials to avoid participants from preparing the button press response already during the presentation of the action sequence. Thus, the spatial position of the words (‘yes’ and ‘no’) on the screen instructed subjects how to respond on any given trial. After the participant responded, a blank screen was presented for a variable interval between 3500 and 4500 ms, upon which the next trial was initiated. A schematic overview of a trial followed by an object question and by a grip question is represented in Figure 2.

Each of the 42 object pairs was presented 8 times according to the Object (Correct vs. Incorrect) x Grasp (Correct vs. Incorrect) x Location (Ipsilateral vs. Contralateral) design, resulting in a total of 336 trials. Care was taken that for each object pair half of all trials were followed by an object question and half of all trials by a grip question. To this end, per subject for each object pair ipsilateral and contralateral pictures were randomly assigned to either goal or grip questions. To familiarize with the task, at the beginning of the experiment participants performed 10 practice trials, representing object pairs that were not used in the main experiment. The experiment consisted of four blocks of 84 trials each and after each block the participant rested.

### EEG Measurements

The electroencephalogram (EEG) was recorded using 61 active electrodes that were placed in an actiCAP (BrainProducts, Munich, Germany). Electrode positions were based on the M-11 61-Channel-Arrangement, encompassing the same areas as the 10/20 system. Horizontal and vertical EOG were measured with electrodes placed on the outer canthi and above and below the participant’s left eye. All electrodes were referenced to the left mastoid online and re-referenced offline to the linked mastoids. The impedance of the electrodes was kept below 20 kOhm. EEG and EOG signals were amplified using two 32-channel BrainAmp DC EEG amplifiers. The signal was sampled at 500 Hz and filtered online with a 125 Hz high cut-off filter and a 10 second time-constant.

The experiment was conducted in an electrically and sound-shielded room. The experiment was controlled by a PC running Presentation software (Neurobehavioral systems Inc., Albany, CA). Markers for the different events were sent to the EEG computer and stored for offline analysis.

### Data Analysis

Analysis of behavioral responses focused on error rates (because of the delayed response paradigm reaction time data were not analyzed). Behavioral data were analyzed using a 2×2×2 repeated measures general linear model (GLM) with Question (Object, Grip), Object (correct, incorrect) and Grip (correct, incorrect) as within-subject variables.

The EEG data was filtered offline using a 30 Hz low-pass filter and 0.1 Hz high-pass filter. For the analysis of event-related potentials ERPs were calculated relative to the onset of the second picture from -200 to 1000 milliseconds using a 100 ms pre-stimulus baseline. Previous EEG studies using a sequence of action pictures have used the image preceding the critical target picture as a baseline as well [9], [10], [22].

Trials with eye movements and movement artifacts were excluded from analysis on the basis of an automated procedure. To test for statistical significance, ERP data was exported in 20 ms bins for each individual subject and per condition across electrodes of interest. Over central sites a 3×3 electrode grid was projected that was analyzed with a 2×2×3×3 repeated measures general linear model (GLM) with Object (correct, incorrect), Grip (correct, incorrect), Anterior-to-Posterior (3 levels) and Left-to-Right (3 levels) as within-subjects factors. To control for multiple comparisons, a criterion of 3 consecutive intervals showing a significant effect was adopted. As 50 intervals were tested, there was a chance of 50×0.05 = 2.5 that one of the intervals shows an effect. By using the criterion of three consecutive significant intervals, this chance is reduced to (50×0.05^3^) = 0.00625, a value lower than significance criterion p = .05 (for a similar statistical approach, see: [3], [5]).

Finally we were interested to what extent participants made systematic eye movements in relation to the detection of object and grip errors. Therefore we analyzed the HEOG (i.e. squared difference between left and right EOG) and the VEOG (i.e. squared difference between the upper and the lower EOG) signals using a 2×2 repeated measures general linear model (GLM) with Object (correct, incorrect) and Grip (correct, incorrect) as within-subjects factors. We applied the same significance criterion as for the main analysis of the ERP data.

## Results

### Behavioral Results

**Figure 3:**
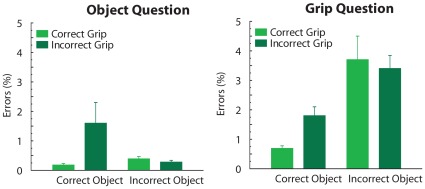
Behavioral results. Error rates in response to object questions (left graph) or to grip questions (right graph). Bars on the left represent responses to action sequences representing grasping of the correct object, bars on the right represent responses to action sequences representing grasping of the incorrect object. Light bars represent responses to action sequences representing a correct grip, dark bars represent responses to action sequences representing an incorrect grip.

Error rates are represented in Figure 3. A main effect of question, F(1, 13) = 220.6, p<.001, η^2^ = .94, reflected that participants made more errors in response to questions about the grip of the action (average percentage of errors = 2.4, SE = .19) compared to questions about the object (average percentage of errors = .58, SE = .20). A main effect of object, F(1,13) = 27.8, p<.001, η^2^ = .68, reflected overall more errors for incorrect compared to correct objects. Finally, an interaction between question and object, F(1,13) = 23.6, p<.001, η^2^ = .65, reflected that for object questions the number of errors was comparable between correct and incorrect objects, whereas for grip questions participants made more errors if the incorrect object was grasped instead of the correct object (see Figure 3).

### Event-related Potentials

**Figure 4:**
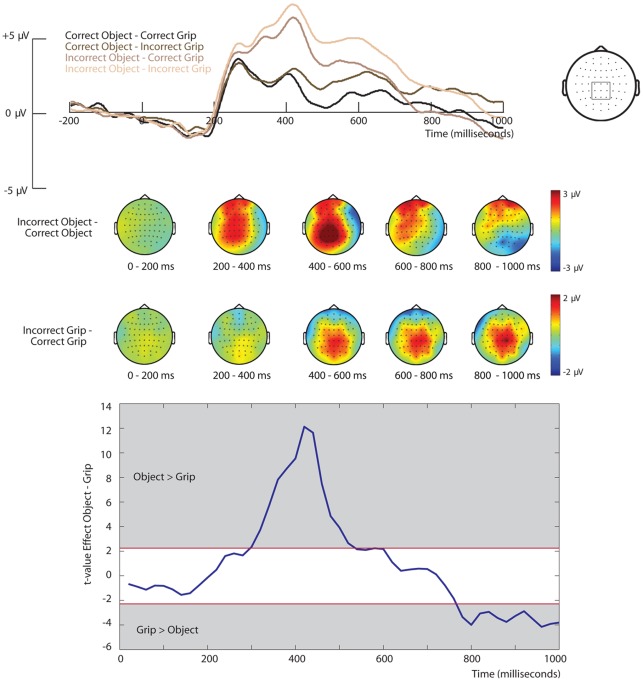
ERPs relative to the onset of the 2^nd^ picture. ERPs relative to the onset of the 2^nd^ picture for a selection of central electrodes. Topographical plots represent the difference between Incorrect and Correct Objects (upper panel) and the difference between Incorrect and Correct Grips (middle panel. The lower panel reflects the t-values for the comparison between the Object Effect (Incorrect – Correct Object) and the Grip Effect (Incorrect – Correct Grip). The critical t-values are marked in red and a positive t-value reflects a stronger effect of Object than of Grip and a negative t-value reflects a stronger effect of Grip than of Object.

The ERPs relative to the onset of the second picture are represented in Figure 4. As can be seen, the onset of the picture representing incorrect action sequences resulted in a P3 effect that developed into a late positivity.

A main effect of Object was found significant from 200 to 720 ms, F(1,13) >4.7, p<.05, reflecting a stronger P3 that developed into a slow wave effect for incorrect compared to correct objects (see Figure 4). An interaction between Object and Anterior-to-Posterior was found significant from 160 to 580 ms, F(2, 26) >4.4, p<.05 and from 600 to 1000 ms, F(2, 26) >5.2, p<.05, reflecting that the difference between incorrect and correct objects was strongest over posterior sites. In addition, an interaction between Object and Left-to-Right from 260 to 1000 ms, F(2, 26) >4.5, p<.05 reflected that the effect of Object was slightly lateralized to the left hemisphere. A main effect of Grip was found significant from 360 to 1000 ms, F(1,13) >4.9, p<.05, reflecting a stronger P3 and a subsequent slow wave effect over central sites for incorrect compared to correct grips (see Figure 4). No interaction between Object and Grip was observed.

To investigate whether the main effects of Object and Grip differed, for each subject and each time bin the averaged effect of Object (Incorrect – Correct Object) and the averaged effect of Grip (Incorrect – Correct Grip) was calculated. Subsequently, these effects were directly compared using a paired-samples t-test. The resulting t-value indicated whether the effects differed significantly. As can be seen in the lower part of Figure 4, from 300 to 520 ms the effect of Object was stronger than the effect of Grip and from 740 to 1000 ms the effect of Grip was stronger than the effect of Object.

### Ocular Movements

For the HEOG a main effect of Object was found significant from 240 to 920 ms, F(1, 13) >4.7, p<.05, and reflected more horizontal eye movements for incorrect compared to correct objects. No main effect of Grip was found, indicating that no overt horizontal eye movements were made for correct compared to incorrect grips. No effects were found for the VEOG, indicating that the observation of object or grip errors did not result in systematic vertical eye movements. No significant interactions were observed.

## Discussion

The aim of the present study was to investigate the functional significance of the P3- and positive slow wave effect associated with processing the correctness of observed actions [9], [10]. It was found that the observation of actions comprising an object-violation resulted in a stronger P3 component and a subsequent parietal positive slow wave effect compared to actions representing a grip-violation.

At a functional level, the P3-effect for the processing of object errors likely reflects an orienting response, directing attention to the unattended object or object part. Whereas many studies have associated the P3-effect with stimulus-driven attention and the detection of deviant or novel stimuli [23], the P3 reflects the evaluative aspect of the orienting response as well [11], [12]. For instance, it has been shown that the P3 effect in response to oddball stimuli is modulated by stimulus familiarity, suggesting that it reflects a relatively late stage of attentional processing, incorporating semantic information [11]. In the present study, based on the functional relation between the objects presented in the first picture, subjects probably generated a strong action prediction about which object would be grasped first and about the grip used for grasping. Observation of actions that did not match this expectation resulted in a stronger P3, likely reflecting a process of stimulus evaluation and spatial reorienting (i.e. directing attention to the other object in case of an object error or the other object part in case of a grip error). This interpretation is supported by the eye movement data, indicating that the detection of object errors was associated with saccadic eye movements to the correct object location. Importantly, the observed P3- and slow-wave effects for object and grip violations preceded the shifts in horizontal eye movements, thereby making it unlikely that these effects can be attributed to mere eye movements. The posterior scalp distribution of the P3 effect provides an additional argument for the notion that this effect cannot be attributed to eye movements. These findings suggest that the detection of action errors, as reflected in the P3 effect precedes the overt redirecting of attention.

Following the P3 effect, the ERPs were characterized by a subsequent parietal positive slow wave effect, similar to the late parietal positivities observed in previous studies on action observation [9], [10]. It has been suggested that the late positivity reflects an evaluative process in which object affordances are evaluated with respect to the preceding action context [10]. Recent studies have reported a comparable parietal positive slow wave effect in association with the execution and online monitoring of real-world actions, that was found maximal at the moment of object grasping [13], [14]. Accordingly, the parietal positive slow wave could reflect the detection of an incorrect hand-object interaction. This interpretation is in line with the functional characteristics of parietal areas that are involved in representing grip-related information [14], [24].

In the present study, the effects of action correctness on the positive slow wave effect were found to be additive and in contrast to previous studies [16] no interaction was found between object- and grip-violations. The absence of an interaction effect is likely due to the experimental design, in which subjects were required to attend to both object- and grip-related aspects of the action at the same time. Therefore participants had to anticipate an appropriate grip for both the tool and the target object and as a consequence effects of grip-violations were observed both for correct and incorrect objects. In contrast, in daily life people probably make an action prediction only about the correct object and not about the incorrect object.

The finding that the P3-effect had an earlier onset and was stronger for goal- compared to grip-violations, suggests that object violations were easier to detect, because the spatial features were more salient for object violations compared to grip violations. Classical studies on visual attention have shown an advantage of space-based attention (i.e. allocation of attention between objects) over object-based attention (i.e. allocation of attention within objects; [25], [26], [27]). Similarly, in the present study the detection of object errors likely required a shift of attention to the other object whereas the detection of grip errors required a shift of attention to a different location within the object. In addition, the temporal precedence of processing object- over grip-related information is directly related to the fact that in order to determine the correctness of the grip applied to an object, one first needs to process which object was grasped. This temporal dependence of object- over grip-related is in line with the hierarchical view of the motor system, according to which the way in which an object is grasped is determined by its consecutive use [18], [19]. Several studies have provided evidence for the hierarchical view, showing for instance a more effective planning process when actions are planned based on object-information compared to grip-related information [14], [24], [28]. The present study extends these findings to the observation of object-directed actions and provides direct neurophysiological evidence for a precedence of processing object- over grip-related information.

Interestingly, the parietal positive slow wave for grip correctness persisted after the effect for object correctness already terminated. It could well be that participants actively maintained a representation of the grip applied to the object until the end of the action sequence, as the correctness of the grip was defined specifically in relation to the subsequent use of the object (e.g. grasping a bubble blower at the opening does not afford bubble blowing). The suggestion that grip errors were more difficult to detect and therefore needed to be actively maintained is further supported by the error data, indicating that subjects made more errors when asked about the correctness of the grip applied to the object than when answering a question about the correctness of the object that was grasped.

### Conclusions

The main finding of the present study is a stronger P3 for the observation of object-violations, likely reflecting a reorienting mechanism, directing attention to the relevant location. A subsequent parietal positive slow wave was found that persisted for the observation of grip-errors that likely reflects the detection of an incorrect hand-object interaction. Thereby this study provides new insight in the functional and neural dynamics that support the understanding of other’s actions.
